# Effectiveness of acupuncture in treating patients with pain and mental health concerns: the results of the Alberta Complementary Health Integration Project

**DOI:** 10.3389/fneur.2024.1366685

**Published:** 2024-08-06

**Authors:** Mingshan Lu, Sumaiya Sharmin, Yong Tao, Xin Xia, Gongliang Yang, Yingying Cong, Guanhu Yang, Jing Jiang, Yun Xiao, Laura Peng, Joshua Quan, Bentong Xu

**Affiliations:** ^1^Department of Economics, University of Calgary, Calgary, AB, Canada; ^2^Department of Community Health Sciences, University of Calgary, Calgary, AB, Canada; ^3^Alberta College of Acupuncture and Traditional Chinese Medicine, Calgary, AB, Canada; ^4^Huatuo Clinic, Calgary, AB, Canada; ^5^Healing Point Acupuncture Clinic/Classic Acupuncture and Herbal Clinic, Los Altos, CA, United States; ^6^Department of Specialty Medicine, Ohio University, Athens, OH, United States; ^7^Department of Forest and Conservation Sciences, Faculty of Forestry, University of British Columbia, Vancouver, BC, Canada

**Keywords:** acupuncture, integrative medicine, pain, mental health, clinical outcomes

## Abstract

**Background:**

This study presents real-world evidence on the clinical outcomes of the Alberta Complementary Health Integration Project (ABCHIP), which utilized acupuncture to address pain and mental health issues in two vulnerable populations in Alberta: youth (aged 24 and below) and elderly (aged 55 and above).

**Methods:**

Over 282 days, a total of 606 patients received 5,424 acupuncture treatments. Tailored to each patients’ specific pain and mental health concerns, an individualized treatment plan was selected, following a standard treatment protocol lasting 1 to 3 months. Patients were evaluated at least twice: initially and upon completing therapy. Primary treatment outcomes were assessed using various measures, including the Brief Pain Inventory (BPI), Pittsburgh Sleep Quality Index (PSQI), Patient Health Questionnaire 9 (PHQ9), PROMIS Anxiety 8a and its pediatric form PROMIS Anxiety-Pediatric, PROMIS Short Form v1.0 Fatigue 8a and its pediatric counterpart PROMIS Pediatric Short Form v2.0 Fatigue 10a, PROMIS Short Form v1.1 Anger 5a and its version PROMIS SF v2.0 5a, and EQ-5D-5L. These measures gauged pain reduction, improved sleep quality, reduced depression, anxiety, fatigue, anger, and quality of life, respectively.

**Results:**

Analysis of data from 500 patients who received at least 6 acupuncture sessions through ABCHIP showed statistically significant improvements in clinical outcomes. Among this group, the subgroup of 235 patients who received at least 12 sessions demonstrated the most favorable treatment outcomes, including an 75.5% reduction in pain severity, a 53.1% improvement in sleep quality, a 78.4% drop in depression, a 41.1% decline in anxiety, a 43.7% decrease in fatigue, a 38.2% decrease in anger, and a 42.6% improvement in overall quality of life.

**Conclusion:**

Integrating acupuncture with usual care demonstrates promise in enhancing mental health, alleviating chronic and general pain, and improving overall quality of life. The findings suggest that integrative programs, such as ABCHIP, present an effective approach to addressing pain and mental health concerns in vulnerable populations, providing valuable insights for future healthcare interventions.

## 1 Introduction

Following the global outbreak of COVID-19, the prevalence of mental health problems has surged due to the broader societal impact and public health responses, including infection control, physical distancing, and quarantine (1, 2). Concerns about mental health and psychosocial well-being, encompassing depressive symptoms, anxiety, stress, post-traumatic stress symptoms, sleep problems, and other psychological disorders, have grown during the COVID-19 pandemic (3). The pandemic’s direct consequences, such as fear of transmission and a sense of danger, have contributed to these issues. Moreover, economic and financial hardships have indirectly impacted mental health (4, 5). The economic shutdowns resulting from the pandemic have had a disastrous impact on global economies, especially in nations with frequent domestic epidemics, inadequate healthcare systems, and high economic vulnerability (6).

Furthermore, chronic pain following an acute COVID-19 infection has exacerbated the situation. There are speculations that the infection might have caused neuroinflammation, a peripheral and central inflammatory response potentially causing persistent musculoskeletal issues and cognitive impairment (7, 8). Chronic weariness, decreased physical ability, and muscle weakness are just a few of the enduring clinical consequences associated with both Middle East respiratory syndrome (MERS) and severe acute respiratory syndrome (SARS) (9). A general decline in the quality of life has been observed long after significant coronavirus epidemics (7–9).

Interest in acupuncture has been steadily growing as individuals and healthcare professionals explore additional ways to manage pain, mental health, addiction, and various chronic health issues. Acupuncture, well-established as a safe and effective adjunct intervention, has been found to significantly improve people’s quality of life and promote overall wellness when integrated with conventional treatments (10–12). Following a holistic approach, acupuncture not only helps alleviate various issues such as chronic pain, mental stress, anxiety, depression, and non-medicated pain relief but also serves as a powerful preventive form of care, strengthening immunity (13–17).

Funded by the government of Alberta, the ABCHIP project provided free acupuncture to address pain, mental health, and addiction issues for youth and elderly in Alberta. Aimed at promoting psychosocial well-being and resilience for these vulnerable populations, ABCHIP sought to mitigate, prevent, and treat pain, mental health, and behavioral issues arising from the COVID-19 pandemic. The project also aimed to reduce dependence on habit-forming pharmaceuticals and promote the integration of acupuncture to deliver patient-centered care.

## 2 Materials and methods

### 2.1 Hypothesis and objective of the study

The study tested the following hypothesis: Patients who received acupuncture would experience improved mental and physical well-being, as well as a higher quality of life.

The objectives of the study were as follows: (1) to measure pain severity before and after the course of treatment; (2) to examine changes in pain interference and physical function; (3) to assess changes in the psychosocial well-being of participants.

### 2.2 Participants

Inclusion criteria were: (1) Youth (aged 24 and below) and the elderly (aged 55 and above), experiencing mental health issues; (2) Those who have any of the following concerns or conditions: mental health concerns and/or conditions (e.g., sleep disorders, anxiety, depression, oppositional defiant disorder, developmental disorders, eating disorders, cognitive impairment and dementia, digestive complaints, etc.); (3) Chronic pain or pain management issues; (4) Addiction (drugs and others).

Exclusion criteria included: (1) Participants refusing to provide their consent; (2) Children whose parents or guardians refused to offer their consent; (3) Patients who revoked their consent; (4) Patients not accessible or comfortable receiving treatment; (5) No-shows without giving notice twice or more.

### 2.3 Recruitment

The study, conducted at the Alberta College of Acupuncture & Traditional Chinese Medicine (ACATCM)—Huatuo Clinic, recruited participants through various channels. Primary care physicians, Alberta Health Services (AHS) mail-out services, and public outreach initiatives served as referral sources. The study also collaborated with primary care doctors to improve patient referrals.

To broaden outreach, the AHS mail-out service, in partnership with the AHS/SPOR group at the Center for Health Informatics (CHI) at the University of Calgary, was utilized. The Enterprise Data Warehouse (EDW) within Alberta Health Services helped identify potential participants in the youth and elderly categories with mental health issues. Advertisements, including project flyers and roadside posters in English, Chinese, and Korean, were employed to attract participants. Individuals could opt for self-referral or receive recommendations from medical professionals.

For recruitment and information dissemination, the study maintained an updated website at http://www.abchip.ca and utilized social media platforms, including Facebook. This multi-faceted recruitment strategy aimed to maximize outreach and ensure diverse participation in the study.

In all the recruiting channels, individuals interested in participating in ABCHIP were directed to submit their applications on the study website. The applications were then reviewed by the project coordinator. All applicants who fit the selection criteria were admitted into ABCHIP to ensure maximum autonomy. Given the limited timeframe of the CHIP program and its nature as a community service program, the recruitment was not done on a random basis. We continued to admit applicants until the end of the one-year program.

Instead of pre-determining a sample size, we continued to admit applicants into ABCHIP throughout the one-year period of this program for the following two reasons:

First, according to our power calculation, the minimum sample size needed for an observational study with 50 predictors would be 450–500 to reach 80% power and a 0.05 significance level. We used this as a minimum threshold for ABCHIP recruitment.

Second, CHIP was a community service program funded through the Government of Alberta’s investment aimed at enhancing mental health and addiction support for Albertans during the COVID-19 pandemic. Maximizing the number of individuals served in the program, subject to the project budget, was one of the program objectives.

At the end of our study, a total of 606 individuals were admitted into CHIP.

### 2.4 Treatment

Patients received effective and complimentary acupuncture treatments from licensed and experienced practitioners with 5–15 years of practice. The primary goal of these treatments was to improve patients’ mental health and well-being by addressing concerns such as pain management, sleep quality, dietary habits, anxiety, and depression. The acupuncture treatment protocols in this study were designed based on established evidence and clinical expertise from local and international leading experts in our team, ensuring that only standard, proven acupuncture treatments were provided, without any experimental procedures.

During the initial consultation, practitioners engaged in discussions with patients and their family members to understand their expectations and treatment goals. Subsequently, they formulated individualized treatment plans for each patient, drawing upon gathered information and discussions, while also referring to established treatment protocols. This approach aligns with the ABCHIP’s guiding principle of providing accountable and patient-centered care. By delivering tailored acupuncture treatments that address the unique needs of each patient, the program aims to offer holistic care for overall health improvement.

Treatment plans were customized based on the patient’s condition and severity, typically lasting 1 to 3 months. Given the community service nature of the project, the goal was to assist as many participants as possible while maintaining a reasonable level of treatment effectiveness. The treatment was conducted twice a week, a frequency proven crucial for optimal results, especially in addressing chronic pain and mental health issues (18–21). In each treatment, a minimum of six acupuncture sessions was the standard. The duration of treatment varied based on the types and severities of patients, with the majority completing their acupuncture regimen within 12 sessions. Some complex cases required additional sessions, but the total did not exceed 18 sessions. Participants could conclude their treatment after the sixth session if they felt their treatment goals were achieved.

### 2.5 Data collection

Patients were evaluated at least twice throughout the study: once at the beginning and once after completion of therapy.

Questionnaires included instruments to measure pain, sleep quality, depression, anxiety, fatigue, anger, and general quality of life, along with demographic questions (age, gender, race/ethnicity, income, etc.) in the baseline surveys. The secure web program REDCap (Research Electronic Data Capture) was utilized for creating and administering online surveys and databases to collect and preserve this data.

After every three treatment sessions, participants were asked to respond to questions from an online questionnaire on REDCap. Information on chronic pain, mental health, and quality of life was again collected for the purpose of treatment effect monitoring and outcome evaluation.

### 2.6 Outcome measures

#### 2.6.1 Pain

The degree of pain and its impact on functioning were evaluated using the Brief Pain Inventory (BPI). The severity of pain was determined by averaging responses to four questions on pain intensity—pain at its “worst” in the previous week, pain at its “least” in the previous week, pain at its “average” in the previous week, and current pain. Pain interference was quantified by averaging responses to seven questions that assessed how much pain affected daily activities: general activity, mood, walking capacity, normal work (both inside and outside the home), relationships, sleep, and enjoyment of life. A higher score on the scale, which ranged from 0 to 10, indicated greater pain severity (22). A scoring system was used, averaging responses to pain severity or interference sections on the BPI. A score of less than 2 indicated none to mild pain, 2–5 indicated moderate pain, and 6 or greater indicated severe pain (23).

#### 2.6.2 Sleep

The Pittsburgh Sleep Quality Index (PSQI) was utilized to assess sleep quality. Component scores, including subjective sleep quality, sleep latency, sleep duration, sleep efficiency, sleep disturbances, use of sleep medication, and daytime dysfunction, were derived from 19 questions according to scoring instructions. The global sleep quality score, representing the sum of these seven components on a scale of 0 to 21, indicated overall sleep quality. PSQI scores exceeding five indicate poorer sleep (24).

#### 2.6.3 Depression

The level of depression was evaluated using the Patient Health Questionnaire 9 (PHQ9). A PHQ9 score was computed by summing responses to nine questions that assessed the frequency of depressive symptoms. A higher PHQ9 score indicated more severe depression, with scores ranging from 0 to 27. A PHQ-9 score of 5 or less indicated no or minimal depression, 5 to 9 indicated mild depression, 10 to 14 indicated moderate depression, and 15 or more indicated severe depression (25).

#### 2.6.4 Anxiety

To measure anxiety in adults, PROMIS Anxiety 8a was employed, while PROMIS Anxiety-Pediatric was utilized for assessing anxiety in children. A final score, ranging from 8 to 40, was derived by summing up eight questions on fear, anxious misery, and hyperarousal. A higher score indicated more severe anxiety. For anxiety scores, values between 0 and 17 indicated no to minimal anxiety, between 17 and 21 suggested mild anxiety, between 22 and 31 indicated moderate anxiety, and scores of 32 and higher were indicative of severe anxiety (26).

#### 2.6.5 Fatigue

The PROMIS Short Form v1.0 Fatigue 8a assessed fatigue in adults, while PROMIS Pediatric Short Form v2.0 Fatigue 10a evaluated fatigue in minors. The total of questionnaire responses produced a fatigue score up to 40, with a higher score indicating greater fatigue. Fatigue values of less than 22 indicated none to slight fatigue, 22–27 suggested mild fatigue, 27–36 indicated moderate fatigue, and a score greater than 36 signified severe fatigue (27).

#### 2.6.6 Anger

Adults were assessed using PROMIS Short Form v1.1 Anger 5a, while minors were assessed using PROMIS SF v2.0 5a. The total score, ranging from 5 to 25, was calculated by summing responses to five questions, with a higher score indicating more intense anger. Scores less than 13 indicated none to slight anger, 13–15 suggested mild anger, 16–20 indicated moderate anger, and a score greater than 21 signified severe anger (28).

#### 2.6.7 Overall quality of life

Overall quality of life was assessed using EQ-5D-5L. This survey explores patients’ mobility, self-care, daily activities, pain/discomfort, and anxiety/depression levels. A health utility score, ranging from slightly under zero (worse than death) to one (complete health), is derived from the responses to these five aspects, with a higher score indicating better health (29).

### 2.7 Data analysis

Data management and analyses were completed using STATA statistical software (College Station, TX). Demographic characteristics of participants were reported using descriptive statistics. Short-term outcomes were assessed using information from participant questionnaires administered at the beginning (baseline) and completion of the treatment (post-treatment). Mean scores for each treatment outcome were described at baseline and post-treatment with associated 95% confidence intervals (CIs). Patients were evaluated at least twice throughout the study: once at the beginning and once after completion of therapy, the overall mean of individual differences in outcome scores from baseline to post-treatment was reported for each outcome with associated 95% CIs. In addition, percent changes in mean values from baseline to post-treatment were also reported for the overall cohort and stratified by group. Additionally, an economic evaluation of ABCHIP has been conducted, and the results are reported in a companion paper (28).

## 3 Results

### 3.1 Study sample

**Figure 1:**
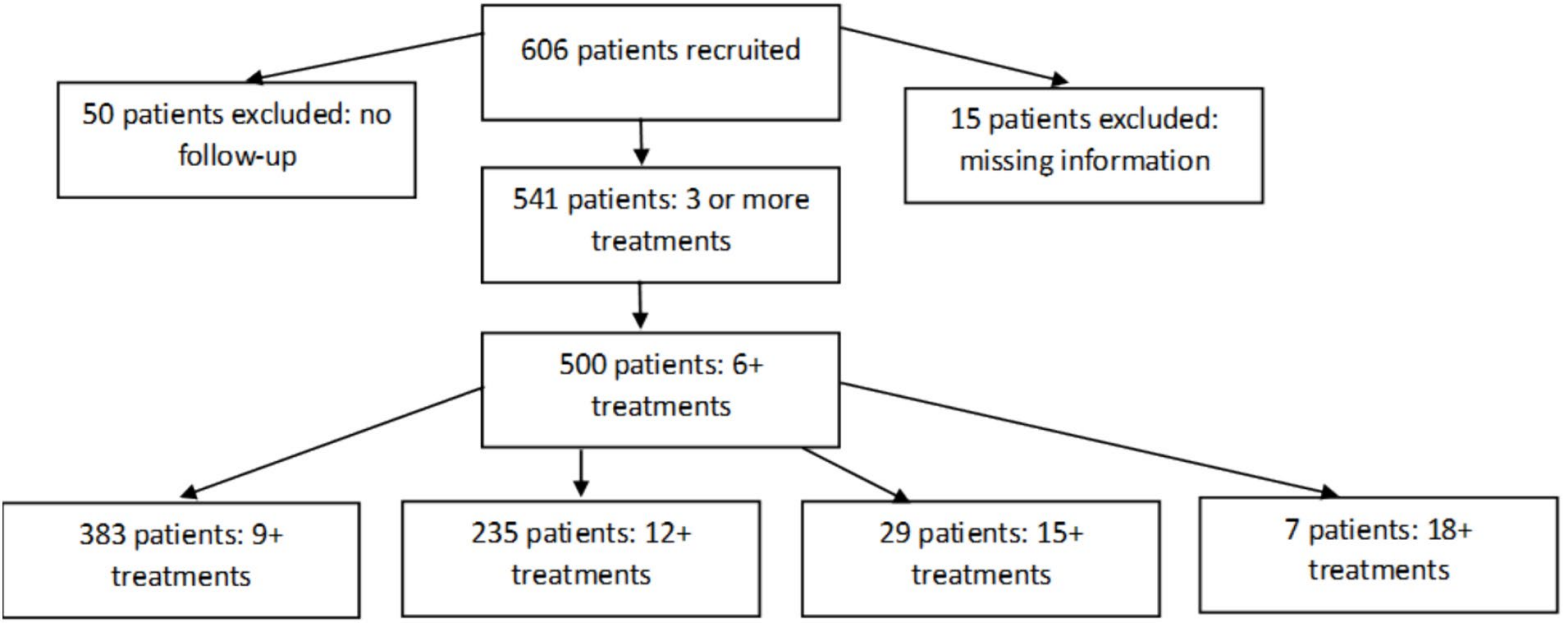
ABCHIP sample.

As depicted in Figure 1, a total of 606 individuals underwent at least one acupuncture session through ABCHIP. Data from 15 patients were excluded due to incomplete questionnaire responses, making interpretation challenging. Furthermore, 91 individuals were excluded as they had undergone fewer than the required minimum of six acupuncture treatments. The majority of patients completed their acupuncture therapy within 12 sessions, except for a few more severe cases that necessitated up to 18 sessions. This yielded a valid sample size of 500.

**Figure 2:**
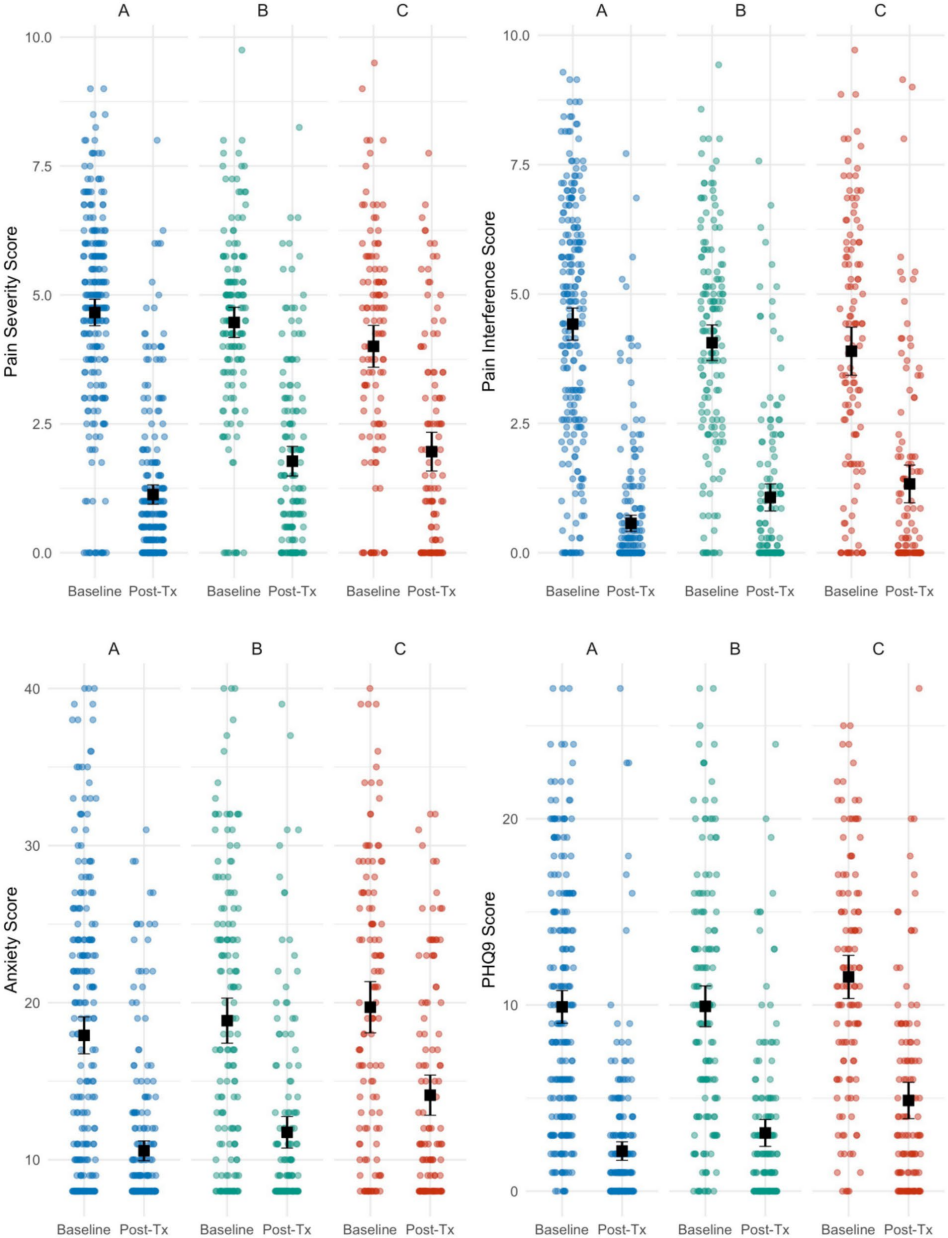

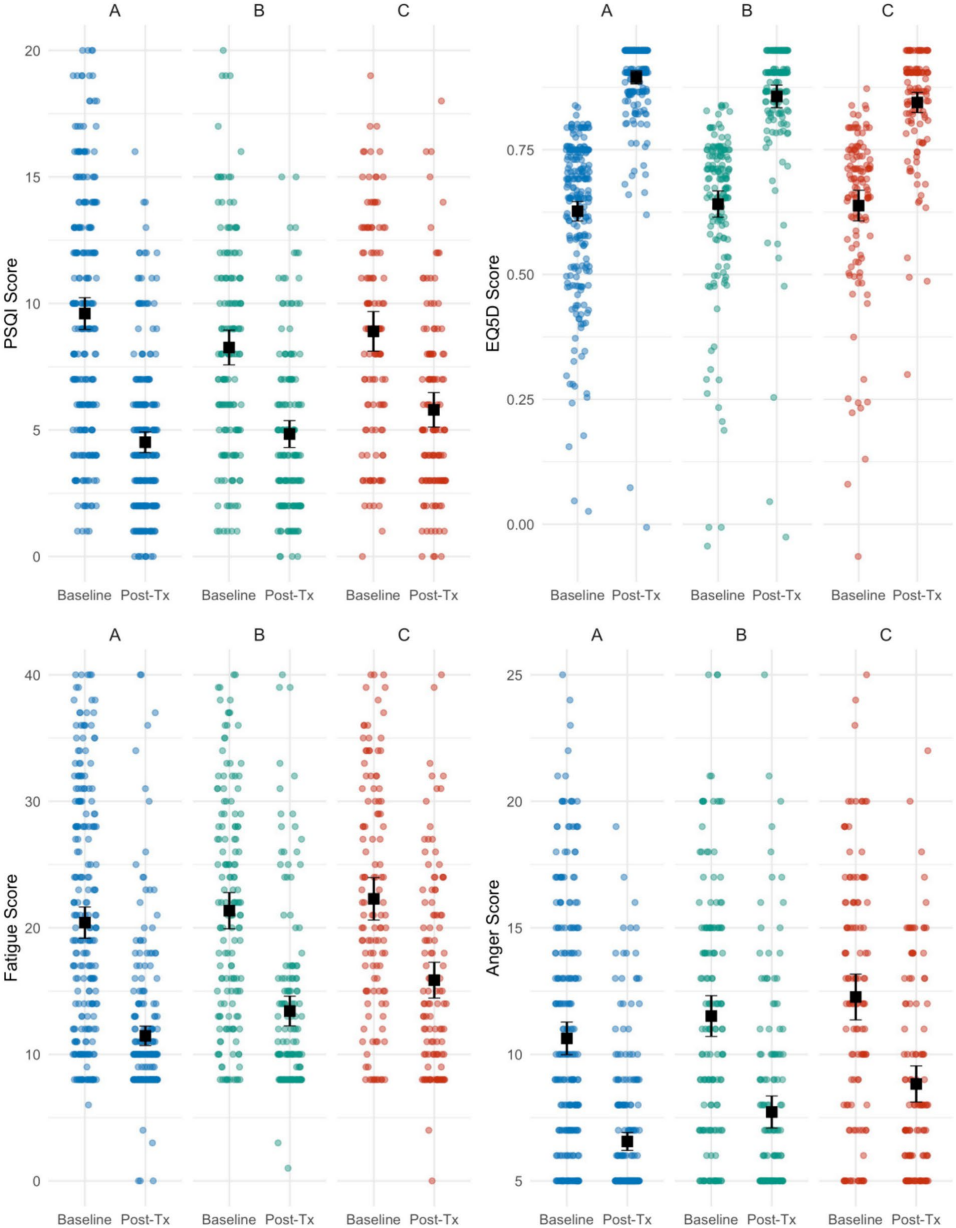
Distribution of scores at baseline and post-treatment by treatment group for each treatment outcome. Black points represent mean value and black error bars represent 95% CIs.

The participants were categorized into three groups based on the total number of acupuncture treatments (Tx) received (Figure 2):

Group A = patients who received a total of 12 or more treatments.
Group B = patients who received a total of 9–11 treatments.
Group C = patients who received a total of 6–8 treatments.

### 3.2 Descriptive characteristics

Table 1 provides a comprehensive overview of the demographic composition of the study sample. Notably, females constitute the majority, representing 73.2% of all respondents. The age cohort from 55 to 74 years up 74.6% of the total. Regarding racial and ethnic backgrounds, East Asians comprise the largest group at 55.4%, followed by Whites at 36.4%. In terms of income distribution, a substantial proportion of respondents fall within the <$13,311 and > $37,801 brackets, accounting for 22.8% and 19%, respectively. Examining marital status, married or common-law individuals form the largest segment, representing 56.2%. Looking at educational attainment, 35.2% of respondents have no post-secondary education, while others hold diverse educational backgrounds, including bachelor’s degrees (16.8%), non-university certificates (12.8%), and graduate degrees (11.6%). Lastly, the findings indicate a significant presence of immigrants in the studied population, with 61.4% identifying as immigrants.

**Table 1:** ABCHIP patient sample demographic characteristics.

Characteristic	Frequency, *n* (%)
**Gender**	
Female	366 (73.2%)
Male	129 (25.8%)
Other	5 (1.0%)
**Age**	
<18 y/o	23 (4.6%)
18–24 y/o	37 (7.4%)
55–64 y/o	222 (44.4%)
65–74 y/o	162 (32.4%)
75+ y/o	36 (7.2%)
No response	20 (4.0%)
**Race/Ethnicity**	
East Asian	277 (55.4%)
White	182 (36.4%)
Mixed Race	12 (2.4%)
South Asian	8 (1.6%)
Southeast Asian	6 (1.2%)
Latin American	5 (1.0%)
Metis	4 (0.8%)
Black	1 (0.2%)
Arab	1 (0.2%)
First Nations	1 (0.2%)
No response	3 (0.6%)
**Income**	
<$13,311	114 (22.8%)
$13,111–$22,628	76 (15.2%)
$22,628–$26,621	53 (10.6%)
$26,621–$37,801	40 (8.0%)
>$37,801	95 (19.0%)
**Marital status**	
Married/Common Law	293 (58.6%)
Single	87 (17.4%)
Divorced/Separated	87 (17.4%)
Widowed	33 (6.6%)
**Highest education**	
No post-secondary education	176 (35.2%)
Bachelor’s degree	84 (16.8%)
Non-university certificate	64 (12.8%)
Graduate degree	58 (11.6%)
Trade/vocational school	44 (8.8%)
Post-secondary cert/diploma	33 (6.6%)
No response	4 (8.2%)
**Student status**	
Junior high	4 (0.8%)
Senior high	19 (3.8%)
University/College	19 (3.8%)
No response	458 (91.6%)
**Immigrant status**	
Immigrant	307 (61.4%)
Non-immigrant	191 (38.2%)
No response	2 (0.4%)
**Main complaint**	
Back pain	99 (19.8%)
Shoulder pain	70 (14.0%)
Neck pain	55 (11.0%)
Knee pain	54 (1.08%)
Depression and anxiety	54 (10.6%)
Sciatica	44 (8.8%)
Anxiety	36 (7.2%)
Depression	30 (6.0%)
Hip pain	16 (3.2%)
Headache	15 (3.0%)
Wrist pain	6 (1.2%)
Leg pain	3 (0.6%)
Osteoarthritis (hand)	3 (0.6%)
Lateral epicondylitis	3 (0.6%)
Addiction	3 (0.6%)
ADHD	2 (0.4%)
Concussion	1 (0.2%)
PTSD	1 (0.2%)

### 3.3 Baseline conditions and treatment outcomes

Distributions of outcome severity and mean outcome scores at baseline and post-treatment are reported in Table 2. Mean individual differences and percent changes in treatment outcomes from baseline to post-treatment are reported in Table 3.

**Table 2:** Distribution of baseline and post-treatment outcomes for all ABCHIP patients.

Outcomes	Baseline frequency, *n* (%)	Baseline mean (95% CI)	Post-Tx frequency, *n* (%)	Post-Tx mean (95% CI)
**BPI group (pain severity)**				
None to mild pain (<2)	52 (10.4%)	4.45 (4.27–4.63)	342 (68.4%)	1.52(1.37–1.67)
Moderate pain (2–5)	295 (59.0%)		134 (26.8%)
Severe pain (6+)	153 (30.6%)	24 (4.8%)
**BPI (pain interference)**				
None to mild pain (<2)	96 (19.2%)	4.19 (3.98–4.40)	415 (83.0%)	0.90(0.76–1.04)
Moderate pain (2–5)	244 (48.8%)		73 (14.6%)
Severe pain (6+)	160 (32.0%)	12 (2.4%)
**PHQ-9 (depression)**				
None (0–4)	124 (24.8%)	10.28 (9.69–10.87)	380 (76.0%)	3.08(2.68–3.48)
Mild (5–9)	117 (23.4%)		82 (16.4%)
Moderate (10–14)	120 (24.0%)	17 (3.4%)
Moderately severe (15–19)	76 (15.2%)	13 (2.6%)
Severe (20–27)	63 (12.6%)	8 (1.6%)
**PROMIS anxiety**				
No to minimal anxiety (0–17)	231 (46.2%)	18.62 (17.83–19.41)	415 (83.0%)	11.75(11.23–12.28)
Mild anxiety (17–21)	69 (13.8%)		33 (6.6%)
Moderate anxiety (22–31)	148 (29.6%)	48 (9.6%)
Severe anxiety (32+)	52 (10.4%)	4 (0.8%)
**PSQI (sleep quality)**				
Good (0–5)	130 (26.0%)	9.03 (8.63–9.44)	323 (64.6%)	4.91(4.61–5.20)
Poor (>5)	370 (74.0%)		177 (35.4%)
**PROMIS fatigue**				
No to slight fatigue (<22)	273 (54.6%)	21.15 (20.34–21.97)	438 (87.6%)	13.07(12.46–13.69)
Mild fatigue (22–27)	67 (13.4%)		32 (6.4%)
Moderate fatigue (27–36)	118 (23.6%)	21 (4.2%)
Severe fatigue (36+)	42 (8.4%)	9 (1.8%)
**PROMIS anger**				
None to slight (<13)	301 (60.2%)	11.28 (10.84–11.72)	440 (88.0%)	7.44(7.13–7.75)
Mild (13–15)	93 (18.6%)		43 (8.6%)
Moderate (16–20)	92 (18.4%)	14 (2.8%)
Severe (21+)	14 (2.8%)	3 (0.6%)
**EQ-5D-5L quality of life**	—	0.63 (0.62–0.65)	—	0.87 (0.86–0.88)

**Table 3:** Mean individual differences and percent change in outcome values from baseline to post-treatment timepoints, stratified by treatment group.

Outcome	Group	Mean difference (95% CI)	Percent change
BPI (pain severity)	Group A	−3.53 (−3.80, −3.26)	−75.5%
	Group B	−2.69 (−3.01, −2.37)	−60.5%
	Group C	−2.04 (−2.41, −1.67)	−51.0%
	Overall	−2.93 (−3.12, −2.74)	−65.9%
BPI (pain interference)	Group A	−3.85 (−4.14, −3.55)	−87.0%
	Group B	−2.99 (−3.32, −2.66)	−73.9%
	Group C	−2.56 (−2.97, −2.15)	−65.8%
	Overall	−3.29 (−3.49, −3.09)	−78.6%
PHQ9 (depression)	Group A	−7.74 (−8.45, −7.03)	−78.4%
	Group B	−6.80 (−7.62, −5.98)	−68.5%
	Group C	−6.63 (−7.63, −5.63)	−57.7%
	Overall	−7.20 (−7.67, −6.73)	−70.1%
PROMIS anxiety	Group A	−7.35 (−8.32, −6.38)	−41.1%
	Group B	−7.11 (−8.25, −5.97)	−37.9%
	Group C	−5.60 (−6.92, −4.27)	−28.4%
	Overall	−6.87 (−7.51, −6.22)	−37.0%
PSQI (sleep quality)	Group A	−5.09 (−5.62, −4.55)	−53.1%
	Group B	−3.43 (−3.95, −2.92)	−41.7%
	Group C	−3.10 (−3.74, −2.46)	−34.9%
	Overall	−4.13 (−4.46, −3.79)	−45.8%
PROMIS fatigue	Group A	−8.94 (−10.00, 7.89)	−43.7%
	Group B	−8.03 (−9.31, −6.75)	−37.7%
	Group C	−6.43 (−8.02, −4.83)	−28.8%
	Overall	−8.08 (−8.80, −7.36)	−38.2%
PROMIS anger	Group A	−4.07 (−4.64, −3.50)	−38.2%
	Group B	−3.79 (−4.47, −3.11)	−33.1%
	Group C	−3.44 (−4.18, −2.69)	−28.0%
	Overall	−3.84 (−4.22, −3.46)	−34.1%
EQ5D-5 L (quality of life)	Group A	0.27 (0.25, 0.29)	42.6%
	Group B	0.22 (0.19, 0.24)	33.7%
	Group C	0.21 (0.18, 0.23)	32.4%
	Overall	0.24 (0.22, 0.25)	37.5%

#### 3.3.1 Pain severity

The majority of patients experienced moderate to severe pain before undergoing treatment in ABCHIP. Approximately 73% of patients identified pain as their primary health concern, with 90% reporting moderate to severe pain severity at baseline. Overall, mean BPI scores fell from 4.45 (95% CI: 4.27, 4.63) at baseline to 1.52 (95% CI: 1.37, 1.67) post-treatment with an average individual difference of −2.93 (95% CI: −3.12, −2.74) and a percent reduction of −65.8%. Participants in Group A (receiving 12 or more acupuncture treatments) experienced the largest reduction in pain severity of 75.5% while participants in Group C (6–8 treatments) experienced the smallest reduction of 51.0% (Table 3).

#### 3.3.2 Pain interference

Overall, 80% of patients reported moderate to severe pain interference at baseline while only 17% of patients reported moderate to severe pain post-treatment. Pain interference was significantly reduced by 3.29 points (95% CI: 3.09, 3.49) on the BPI scale from baseline (BPI: 4.19; 3.98, 4.40) to treatment (BPI: 0.90; 0.76, 1.04), corresponding to a percent change of 78.5% for the overall cohort (Table 3). Similarly, pain interference was most reduced for Group A, and least reduced for Group C, although all groups reported a significant reduction in pain interference, underscoring acupuncture’s effectiveness in substantially enhancing patients’ quality of life by alleviating pain-related challenges.

#### 3.3.3 Depression

17% of patients expressed overall concern about depression, with 52% reporting moderate to severe depression at baseline and a mean PHQ-9 score of 10.28 (95% CI: 9.69, 10.87). Following treatment, only 7.6% of individuals reported moderate to severe depression with a mean PHQ-9 of 3.08 (95% CI: 2.68, 3.48). PHQ-9 scores were reduced by an average of 7.20 (95% CI: 6.73, 7.67) points following treatment, corresponding to a 70.0% overall decrease (Table 3). Groups A, B, and C experienced 78.4, 68.5, and 57.7% decreases, respectively; all decreases were statistically significant.

#### 3.3.4 Anxiety

18% of patients reported an anxiety disorder, with 40% of participants experiencing moderate to severe anxiety at baseline (mean score: 18.62; 95% CI: 17.83, 19.41). Only 10.4% of participants reported moderate to severe anxiety after treatment (mean score: 11.75; 95% CI: 11.23, 12.28). Anxiety scores significantly decreased for the overall cohort by 36.9% and for each individual treatment group (Table 3).

#### 3.3.5 Sleep quality

Approximately 74% of ABCHIP patients reported poor sleep quality at baseline, while 35.4% reported poor sleep quality post-treatment. For the overall cohort, PSQI scores dropped significantly by 4.13 (95% CI: 4.46, 3.79); there was a 45.6% decrease from PSQI scores at baseline (9.03; 8.63, 9.44) to post-treatment (4.91; 4.61, 5.20). Groups A, B, and C reported 53.1, 41.7, and 34.9% reductions in PSQI scores, respectively. All decreases were statistically significant.

#### 3.3.6 Fatigue

Approximately 67% of patients experienced moderate to severe fatigue at baseline, reporting at mean PROMIS fatigue score of 21.15 (95% CI: 20.34, 21.97). Post-treatment, only 7% of patients reported moderate to severe fatigue and the mean score was 13.07 (95% CI: 12.46, 13.69). Overall fatigue scores dropped by 38.2%; mean differences were significantly negative for the overall cohort (−8.08; 95% CI: −8.80, −7.36) and all treatment groups.

#### 3.3.7 Anger

While 21.2% of participants reported moderate to severe anger scores at baseline, only 3.4% reported following treatment. Anger scores decreased from 11.28 (95% CI: 10.84, 11.72) to 7.44 (95% CI: 7.12, 7.75), corresponding to a 34.0% decrease. The mean individual decrease was by 3.84 points (95% CI: 3.46, 4.22) for the overall cohort; decreases were significantly for all treatment groups.

#### 3.3.8 Overall quality of life

The average EQ5D-5 L score for pain, depression, and anxiety patients was 0.63 (95% CI: 0.62, 0.65) at baseline, lower than the average EQ5D-5 L score for Albertans in 2018, which was 0.85 (30). However, the mean EQ5D-5 L score was significantly higher post-treatment at 0.87 (95% CI: 0.86, 0.88), corresponding to a percent increase of 38.1%. The average individual increase in EQ5D-5 L was 0.24 (95% CI: 0.22, 0.25); this increase was significantly for all treatment groups.

## 4 Discussion

Analysis of data from 500 patients who received at least 6 acupuncture sessions through ABCHIP showed statistically significant improvements in clinical outcomes. Among this group, the subgroup of 235 patients who received at least 12 sessions demonstrated the most favorable treatment outcomes, including an 75.5% reduction in pain severity, a 53.1% improvement in sleep quality, a 78.4% drop in depression, a 41.1% decline in anxiety, a 43.7% decrease in fatigue, a 38.2% decrease in anger, and a 42.6% improvement in overall quality of life.

This study has certain limitations. Firstly, ABCHIP is not a randomized controlled trial; instead, it focuses on providing community services and real-world evidence. Secondly, all data used for constructing outcome measures and conducting treatment evaluations are self-reported. Despite using instruments with high validity and reliability, these measures are still subject to reporting errors.

Nevertheless, the findings from ABCHIP suggest that integrating acupuncture with usual care demonstrates promise in enhancing mental health, alleviating chronic and general pain, and improving overall quality of life. Integrative programs, such as ABCHIP, offers a holistic approach to addressing both physical symptoms and psychological well-beings. This approach is particularly beneficial in vulnerable populations where conventional treatments may have limitations.

The findings from ABCHIP underscore the potential of integrative medicine to provide comprehensive support for patients experiencing pain and mental health challenges. By integrating acupuncture, a therapy known for its stress-reducing and pain-relieving effects, into conventional care, the program not only addresses symptoms but also promotes overall quality of life. This integrative approach aligns with the principles of psychosomatic medicine, which recognizes the intricate interplay between mental and physical health.

Moreover, our study highlights the importance of personalized and patient-centered care approaches in healthcare interventions. By tailoring treatments to individual needs and incorporating therapies that address both mind and body, healthcare providers can better support patients facing complex health challenges.

In conclusion, the findings from ABCHIP provide valuable insights into the benefits of integrative programs in addressing pain and mental health issues. This study not only supports the integration of complementary therapies into clinical practice but also underscores the relevance of psychosomatic considerations in healthcare delivery. Future research and healthcare interventions can build upon these insights to further optimize patient care and outcomes in diverse patient populations.

## Data availability statement

The datasets presented in this article are not readily available because data requests are subject to ethics approval by the University of Calgary Conjoint Health Research Ethics Board (CHREB). Requests to access the datasets should be directed to CHREB, chereb@ucalgary.ca.

## Ethics statement

The studies involving humans were approved by the University of Calgary Conjoint Health Research Ethics Board (CHREB) (Ethics ID: REB 21-0086). The studies were conducted in accordance with the local legislation and institutional requirements. Written informed consent for participation in this study was provided by the participants’ legal guardians/next of kin.

## Author contributions

ML: Conceptualization, Data curation, Formal analysis, Funding acquisition, Investigation, Methodology, Project administration, Supervision, Validation, Visualization, Writing – original draft, Writing – review & editing. SS: Visualization, Writing – original draft, Writing – review & editing. YT: Conceptualization, Methodology, Writing – original draft, Writing – review & editing. XX: Formal analysis, Investigation, Methodology, Supervision, Writing – original draft, Writing – review & editing. GoY: Project administration, Resources, Visualization, Writing – original draft, Writing – review & editing. YC: Validation, Writing – original draft, Writing – review & editing, Investigation. GuY: Formal analysis, Methodology, Validation, Writing – original draft, Writing – review & editing. JJ: Investigation, Project administration, Writing – original draft, Writing – review & editing. YX: Data curation, Formal analysis, Project administration, Software, Visualization, Writing – original draft, Writing – review & editing. LP: Data curation, Formal analysis, Software, Visualization, Writing – original draft, Writing – review & editing. BX: Conceptualization, Funding acquisition, Investigation, Project administration, Resources, Supervision, Writing – original draft, Writing – review & editing. JQ: Formal analysis, Visualization, Writing – review & editing.
